# The Impact of Nuclear Factor Kappa B on the Response of Microglia in Spinal Cord Injuries

**DOI:** 10.7759/cureus.79367

**Published:** 2025-02-20

**Authors:** Iordanis Varsamos, Christos Patilas, Athanasios Galanis, Dimitrios Zachariou, Georgios Tsalimas, Evangelos Sakellariou, Ioannis Spyrou, Meletis Rozis, Angelos Kaspiris, Panayiotis K Karampinas, Elias Vasiliadis, Spyros G Pneumaticos

**Affiliations:** 1 3rd Department of Orthopedics, National and Kapodistrian University of Athens, KAT General Hospital, Athens, GRC; 2 Division for Orthopaedic Research, Laboratory of Molecular Pharmacology, School of Health Sciences, University of Patras, Patras 26504, Greece, Patras, GRC

**Keywords:** inflammatory response, microglia, nf-kb, nf-kb signaling pathway, spinal cord injury

## Abstract

Spinal cord injury (SCI) results in both primary and secondary damage, each contributing to the overall injury and its consequences. Following SCI, microglia, the resident immune cells of the central nervous system (CNS), undergo a series of complex responses that contribute to the pathophysiology of the injury. In the context of SCI, nuclear factor kappa B (NF-kB) emerged as a critical mediator in the regulation of inflammatory responses following SCI. The aim of this review is to provide a comprehensive understanding of the involvement of NF-kB in the response of microglia following SCI.

The PUBMED database was searched using the following keywords: NF-kB AND microglia AND spinal cord injury. Clinical and experimental studies evaluating the role of NF-kB in the response of microglia following SCI were included. Systematic reviews, case reports, research protocols, conference articles, and studies in languages other than English were excluded. The final analysis included 52 studies.

NF-kB signaling exerts profound effects on the microglial response following SCI, influencing the inflammatory milieu, tissue damage, and potential for repair and recovery. Deactivation of the NF-kB signaling pathway suppresses the production of proinflammatory mediators in microglia, after SCI. Moreover, NF-kB suppression has neuroprotective effects, as it mitigates neuronal apoptosis and facilitates the M2 microglial phenotype, alleviating tissue damage after SCI. Moreover, several microRNAs play a crucial role in regulating gene expression post-transcriptionally and have emerged as key regulators in microglia activation after SCI.

Overall, the role of NF-kB in the response of microglia to SCI is complex and context-dependent. While NF-kB activation is involved in initiating and propagating the inflammatory response following SCI, it also plays a role in tissue repair and regeneration. Thus, modulating NF-kB signaling in microglia represents a potential therapeutic target for attenuating inflammation and promoting neuroprotection and tissue repair in SCI.

## Introduction and background

Spinal cord injury (SCI) results in both primary and secondary damage, each contributing to the overall injury and its consequences. Primary damage refers to the initial mechanical injury that occurs at the moment of impact or trauma to the spinal cord. It often involves physical disruption or severing of the spinal cord tissue. Primary damage can result from various mechanisms, including compression, contusion, laceration, or transection of the spinal cord. It may lead to immediate loss of function below the level of injury such as loss of sensation, motor function, and autonomic control. Secondary damage refers to the cascade of pathological processes that occur after the initial injury and exacerbate tissue damage and functional impairment over time. Secondary damage can unfold over minutes, hours, days, or even weeks following the initial injury. It involves complex biochemical, cellular, and molecular events, including inflammation, excitotoxicity, oxidative stress, blood-spinal cord barrier disruption, spinal cord edema, apoptosis, and necrosis. The primary and secondary damage processes interact and influence each other, leading to a complex cascade of events that determine the extent of injury and functional outcomes following SCI [1-3].

Inflammatory responses are triggered following SCI, involving the release of cytokines, chemokines, and immune cells. While inflammation is a crucial part of the body's defense mechanism and tissue repair, excessive or prolonged inflammation can exacerbate tissue damage and contribute to secondary injury. Glutamate, an excitatory neurotransmitter, is released excessively following SCI, leading to overstimulation of neurons and subsequent cell death. Excitotoxicity contributes to neuronal damage and dysfunction after SCI [4-5]. Reactive oxygen species (ROS) are produced in excess following SCI, leading to oxidative damage to cellular components such as lipids, proteins, and deoxyribonucleic acid (DNA). Oxidative stress contributes to cell death and tissue damage after SCI [6-7]. Disruption of the blood-spinal cord barrier occurs following SCI, leading to increased permeability and infiltration of inflammatory cells and molecules into the spinal cord tissue, exacerbating inflammation and tissue damage [8]. Swelling and edema occur at the site of injury and in the surrounding spinal cord tissue, leading to further compression of neural structures and impaired blood flow, exacerbating tissue damage and functional impairment. Programmed cell death (apoptosis) and uncontrolled cell death (necrosis) contribute to neuronal and glial cell loss following SCI, exacerbating tissue damage and functional impairment [9].

Following SCI, microglia, the resident immune cells of the central nervous system, undergo a series of complex responses that contribute to the pathophysiology of the injury. The microglial response to SCI can be broadly characterized into several phases: During the acute phase (from hours to days), microglia become activated rapidly in response to the injury, undergoing morphological changes and upregulating cell surface markers associated with activation such as clusters of differentiation 11b (CD11b) and 68 (CD68). Activated microglia release pro-inflammatory cytokines, including tumor necrosis factor α (TNF-α), interleukin 6 (IL-6), and interleukin-1 beta (IL-1β), contributing to the inflammatory response and secondary injury cascade. Microglia phagocytose cellular debris and apoptotic cells at the site of injury, aiding in the clearance of damaged tissue [10]. During the subacute phase (from days to weeks) microglia change from a predominantly pro-inflammatory phenotype to a mixed phenotype with both pro-inflammatory and anti-inflammatory properties. Microglia contribute to tissue repair and remodeling by promoting angiogenesis, extracellular matrix remodeling, and neurotrophic factor production. Microglia play a role in resolving inflammation by producing anti-inflammatory cytokines and regulating the activity of other immune cells [11]. At the chronic phase (from weeks to months), microglia may remain activated for an extended period, contributing to chronic inflammation and tissue damage. Persistent microglial activation has been implicated in the development and maintenance of neuropathic pain following SCI. At last, microglia contribute to scar formation and glial scar remodeling, which can have both beneficial and detrimental effects on tissue repair and axonal regeneration [12-14].

NF-kB is a transcription factor that plays a central role in regulating the expression of genes involved in inflammation, immune response, cell survival, differentiation, and proliferation. It is involved in a wide range of physiological and pathological processes, including immune response, inflammation, cancer, and neurodegenerative diseases. NF-kB belongs to a family of transcription factors composed of homo- or heterodimers of subunits belonging to the Rel family [15]. The most common form of NF-kB is a heterodimer consisting of p50 and p65 (RelA) subunits. Other subunits include gene p52, Transcription factor RelB, and c-Rel. NF-kB is typically sequestered in the cytoplasm in an inactive form through its association with inhibitory proteins known as inhibitors of nuclear kappa B (IkBs). Various stimuli, such as pro-inflammatory cytokines, microbial products, oxidative stress, and DNA damage, can activate NF-kB signaling pathways. Activation typically involves the phosphorylation and degradation of IkBs, allowing NF-kB to translocate into the nucleus and activate target gene expression [16-18].

NF-kB regulates the expression of a wide range of genes involved in immune and inflammatory responses, including cytokines (e.g., TNF-α, IL-1B, IL-6), chemokines, cell adhesion molecules, and inflammatory enzymes (e.g., cyclooxygenase-2 (COX-2), inducible nitric oxide synthase (iNOS)). NF-kB also regulates genes involved in cell survival (e.g., B-cell lymphoma 2 (Bcl-2), B-cell lymphoma-extra large (Bcl-xL)), proliferation, and differentiation. It plays a central role in orchestrating the expression of pro-inflammatory mediators and immune cell activation in response to pathogens, tissue injury, and other stimuli. Dysregulation of NF-kB signaling has been implicated in various diseases, including inflammatory diseases (e.g., rheumatoid arthritis, inflammatory bowel disease), autoimmune diseases (e.g., multiple sclerosis, lupus), cancer, neurodegenerative diseases (e.g., Alzheimer's disease, Parkinson's disease), and metabolic disorders (e.g., obesity, diabetes) [19-21].

Modulating NF-kB responses in the CNS is an area of active research for potential therapeutic interventions. In the context of SCI, the role of NF-kB becomes particularly intriguing, as it has emerged as a critical mediator in the regulation of inflammatory responses following SCI. The aim of this review is to provide a comprehensive understanding of the involvement of NF-kB in the response of microglia following SCI.

## Review

Material and methods

A literature review was conducted based on the PubMed internet database, with the use of the EndNote X3 software (Thompson Reuters, Toronto, Canada) [22]. Article titles were searched with the use of the keywords: "NF-kB” AND “microglia” AND “spinal cord injury”. Clinical and experimental studies evaluating the role of NF-kB in the response of microglia following SCI were included. Systematic reviews, case reports, research protocols, conference articles, and studies in languages other than English were excluded.

Results

**Figure 1:**
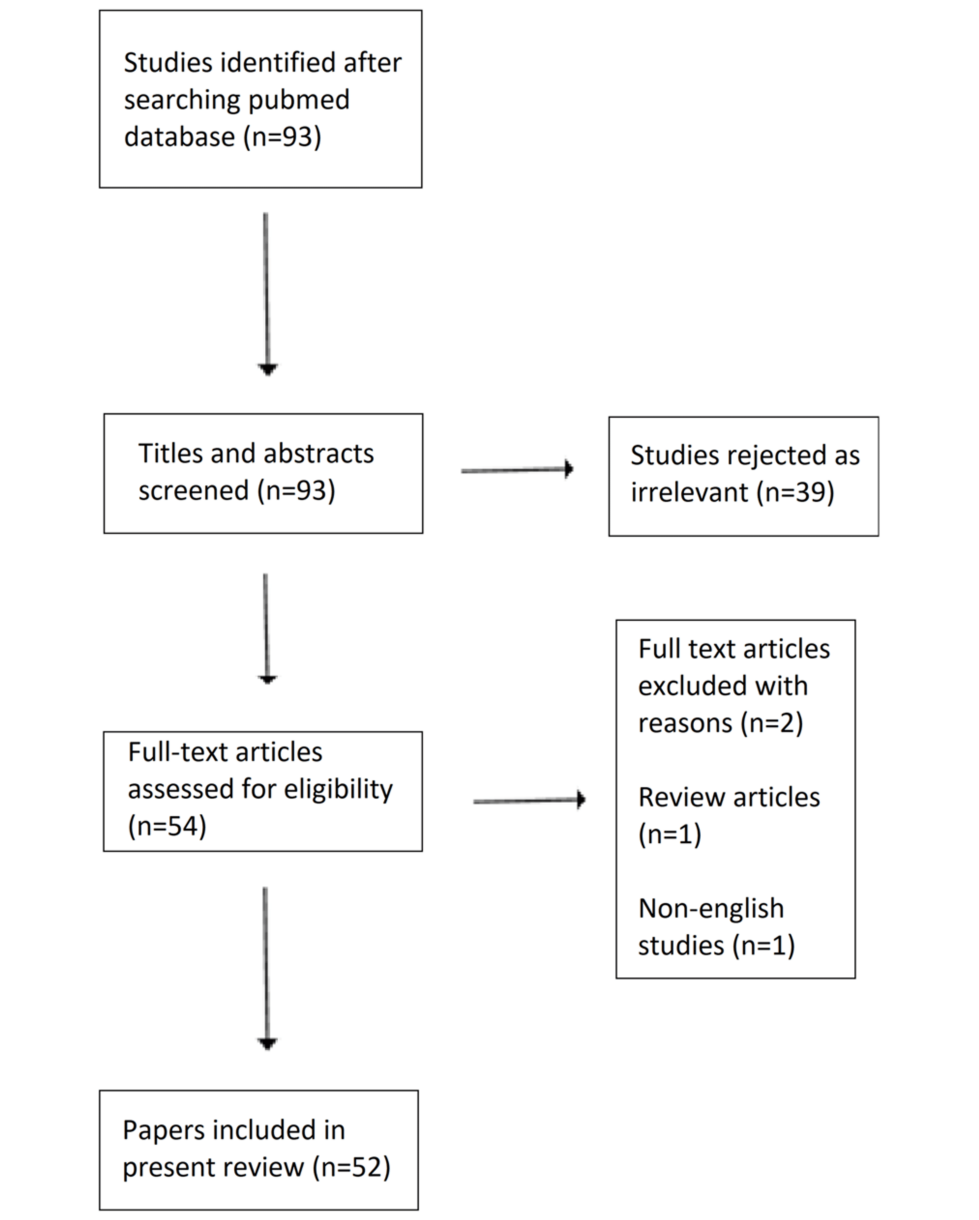
Study flowchart

Initially, 93 studies were identified after a primary search on the PubMed electronic database. After screening titles and abstracts, 39 articles were rejected as not relevant to the study. Among the remaining 54 studies, 2 were excluded for various reasons (Figure 1), leaving 52 studies for final analysis.

Microglia are scavengers that phagocytose cellular debris, myelin fragments, and apoptotic cells to facilitate tissue clearance and repair. Within two hours after SCI, microglia are the first immune cells to be activated and trigger the initiation and progression of the inflammatory response, undergoing morphological and molecular changes, transitioning from a ramified resting state to an amoeboid or hypertrophic phenotype with enlarged cell bodies and retracted processes [23-24]. In experimental studies, activated microglia reach their maximum one week after SCI and gradually decrease after three weeks [25].

**Figure 2:**
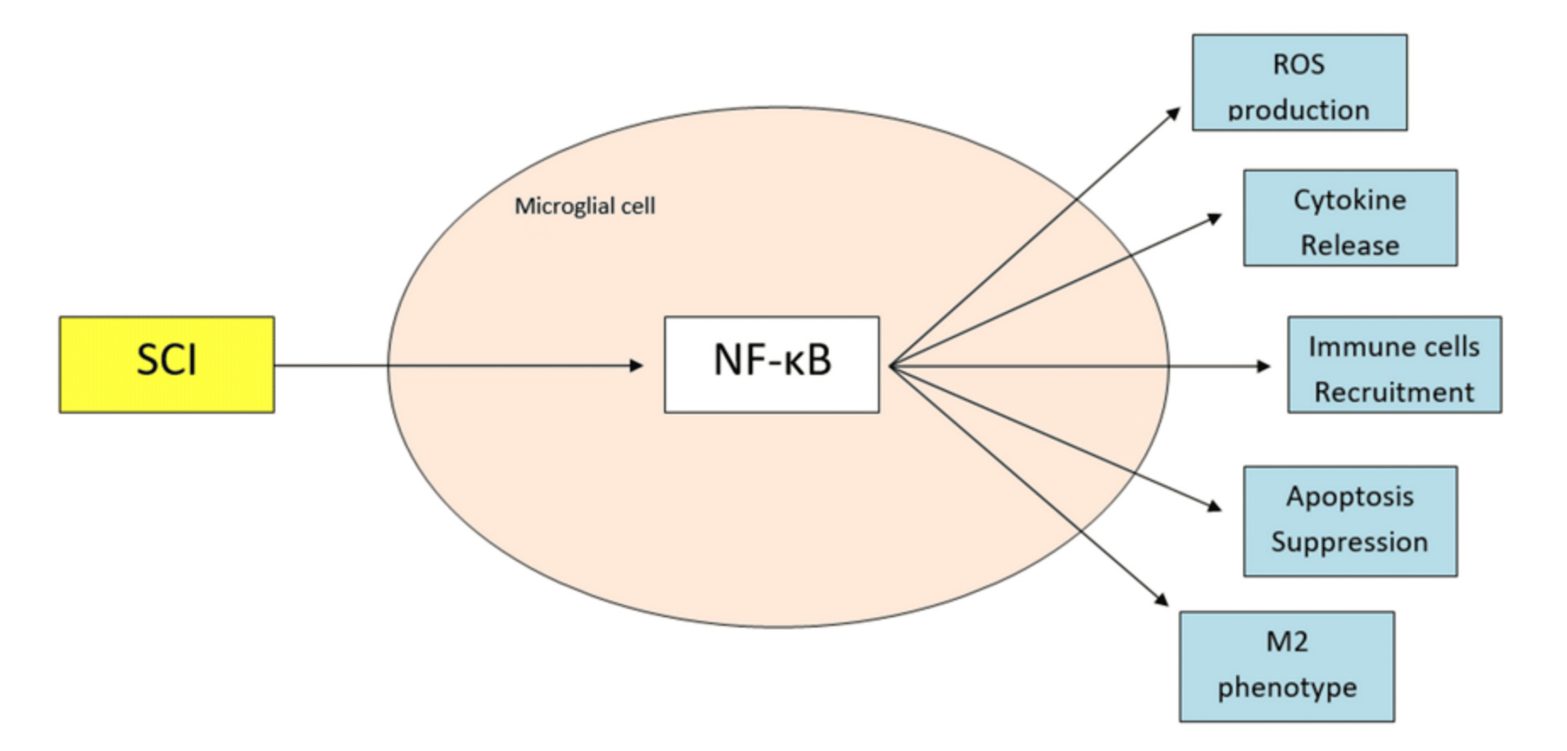
NF-kB overview diagram Original diagram by the authors

Depending on the signals they receive from the environment, microglia can undergo both pro-inflammatory (M1) and anti-inflammatory (M2) polarization. The M1 phenotype is associated with the production of proinflammatory cytokines such as TNF-α, IL-1B, IL-6, NO, and ROS. In contrast, the M2 phenotype is characterized by the production of anti-inflammatory cytokines (IL-4, IL-10, IL-13) and growth factors such as transforming growth factor beta (TGF-B), ciliary neurotrophic factor (CNTF), insulin-like growth factors (IGF), epidermal growth factor (EGF), and nerve growth factor (NGF), which compete the pro-inflammatory responses and facilitate neuroregeneration, particularly axonal extension, after SCI. Following SCI, microglia are predominantly polarized toward the M1 phenotype, which is characterized by the production of pro-inflammatory cytokines, ROS, and NO. In the chronic phase of SCI, microglia are shifted toward the M2 phenotype (Figure 2) [26].

Discussion

Activation of NF-kB in Microglia After Spinal Cord Injury

NF-kB activation is one of the key pathways that induce microglial activation following SCI. Various stimuli associated with SCI such as pro-inflammatory cytokines, damage-associated molecular patterns (DAMPs), and reactive oxygen species (ROS), can activate NF-kB signaling in microglia, leading to their activation and production of inflammatory mediators [27]. The toll-like receptor 4 (TLR4) signaling pathway is vital in activated microglia-induced neuroinflammatory responses, and TLR4 is highly expressed on the microglial membrane after an SCI [28]. In the CNS, lipopolysaccharides (LPS) connect to and activate TLR4, leading to the activation of NF-kB and mitogen-activated protein kinases (MAPKs) [29]. The TLR4/NF-kB pathway in microglia acts as an amplification loop, with pro-inflammatory cytokines further activating microglia and enhancing NF-kB signaling. This sustained activation of microglia perpetuates neuroinflammation and contributes to secondary injury mechanisms, including neuronal cell death, axonal degeneration, and glial scar formation [28]. FGF10 administration suppresses microglia activation and proliferation through the regulation of the TLR4/NF-kB pathway and attenuates the inflammatory response in SCI animals [23].

Animal studies have shown that NF-kB immunoreactivity was detected primarily within and adjacent to the lesion epicenter, at 30 min and 90 min following SCI. At 24 and 72 hours after SCI, NF-kB immunoreactivity was detected throughout the extent of the spinal cord section. Activated NF-kB was detected in macrophages/ microglia, neurons, and endothelial cells and is still present three days after SCI [27].

Inflammatory Effects of Nf-kB Through Microglia After SCI

NF-kB is regarded as the central transcription factor of inflammatory mediators, where it plays a vital role in microglial activation. NF-kB activation in microglia leads to the production and release of pro-inflammatory cytokines and chemokines. Cytokine production is regulated primarily at the transcriptional level. Upon activation, NF-kB translocates into the nucleus and induces the transcription of genes encoding pro-inflammatory mediators such as TNF-α, IL-1B, IL-6, and iNOS. These inflammatory mediators contribute to the secondary injury cascade following SCI, exacerbating tissue damage and neuronal death [27].

Several molecules can affect the inflammatory effects of NF-kB signaling through microglia activation after SCI. These molecules can modulate various steps of the NF-kB signaling pathway, including its activation, nuclear translocation, DNA binding, and transcriptional activity, thereby influencing the inflammatory response mediated by microglia. There are anti-inflammatory molecules, such as curcumin, which inhibit NF-kB activation, leading to reduced production of pro-inflammatory cytokines and chemokines by microglia. Moreover, there are pro-inflammatory molecules, such as HMGB1, Il-1B, TNF-α, and LPS, which activate NF-kB signaling in microglia, leading to the production of pro-inflammatory cytokines and exacerbation of neuroinflammation after SCI. Finally, there are endogenous modulators, such as PPAR-γ, Nrf2, and IL-10, which suppress NF-kB activation and signaling in microglia, leading to reduced inflammation and oxidative stress [27-32].

STAT proteins are a family of transcription factors that play critical roles in transducing extracellular signals from cytokines, growth factors, and other signaling molecules to regulate gene expression. Cross-talk between the STAT and NF-kB pathways may amplify inflammatory responses in microglia and exacerbate tissue damage after SCI. NF-kB activation process co-exists with STATs. The DNA-binding STATs reach the nucleus and activate their target genes including Bcl-3, which encodes elements of IkB from the NF-kB pathway. Valproic acid administration may mitigate the inflammatory response and decrease IL-6 production by regulating microglia polarization through STAT1-mediated acetylation of the NF-kB pathway, dependent on HDAC3 activity. The acetylation status of NF-kB p65 and the complex with NF-kB p65 and STAT1 inhibited the NF-kB p65 transcriptional activity and attenuated the microglia-mediated central inflammatory response following SCI [30].

The stimulator of interferon genes (Sting) pathway is an important component of the innate immune system that senses cytosolic DNA and activates downstream signaling cascades, ultimately leading to the production of type I interferons and other pro-inflammatory cytokines. Sting knockout alleviated inflammatory response and facilitated recovery after SCI in mice through blocking TANK-binding kinase 1 activation and subsequent NF-kB and MAPKs phosphorylation [33]. HMGB2 belongs to a family of DNA-binding proteins that play diverse roles in gene regulation, chromatin structure, and inflammation. Extracellular HMGB2 can activate microglia and other immune cells by binding to specific receptors, such as TLR4 and RAGE, leading to the production of pro-inflammatory cytokines and chemokines. A study by Yang et al. observed that HMGB2 knockdown suppressed the canonical NF-kB signaling pathway both in vivo and in vitro, inhibiting microglia-mediated neuroinflammation after SCI [34].

Several signaling molecules (TREM2, MALT1, SphK1, CD93, TNIP, STIP1, BRD4, TRIM52, CARD6) have been found to suppress the NF-kB signaling pathway, mediating its inflammatory effects in microglia following SCI. TREM2 is a cell surface receptor predominantly expressed by microglia in the CNS. It belongs to the immunoglobulin superfamily and plays a crucial role in regulating microglial function, including its activation state and response to injury and inflammation. Downregulating the expression of TREM2 can attenuate IL-6 release from stimulated microglia by suppressing the NF-kB signaling pathway in SCI patients [35]. MALT1 is a paracaspase enzyme that plays a crucial role in immune signaling and inflammation, particularly in lymphocytes. MALT1 knockdown declines IL-6 expression and inhibits microglia activation, M1 polarization, and neuroinflammation through suppression of NF-kB pathway in SCI. MALT1-mediated activation of NF-kB in microglia may exacerbate neuroinflammation and tissue damage in the injured spinal cord [36]. SphK1 is an enzyme that phosphorylates sphingosine to form S1P, a bioactive lipid mediator involved in various cellular processes, including cell proliferation, survival, migration, and inflammation. An animal study by Wang et al. concluded that the S1P/S1PR3/p38 MAPK pathway in microglia contributes to the NF-kB p65 inflammatory response, after SCI [37]. CD93 is a transmembrane glycoprotein that belongs to the C-type lectin domain family. While CD93 is primarily known for its role in endothelial cells, where it regulates angiogenesis and vascular development, its expression has also been reported in other cell types, including immune cells such as macrophages and microglia. Hong et al. found that CD93 expression increased in microglia after SCI in vivo or after LPS stimulation in vitro. Additionally, CD93 interacted with TAK1 to inhibit NF-kB activation, thus suppressing inflammation and microglial migration after LPS stimulation [38]. Additionally, TNIP2 is a protein that downregulates NF-kB signaling, as it may connect to A20 and attenuate inflammatory mediators-induced NF-kB activation. A recent study by Fu et al. observed that in SCI rats, TNIP was highly expressed leading to the inhibition of M1 polarization and production of TNF-α, IL-1B, and IL-6 in microglia, through the suppression of the NF-kB signaling pathway [39]. STIP1 is a co-chaperone protein involved in the regulation of protein folding and cellular stress responses. STIP1 has been implicated in modulating immune responses and inflammatory signaling pathways. It interacts with NF-kB and affects the intensity and duration of microglial activation and the subsequent inflammatory cascade following SCI. STIP1 alleviates ischemia/reperfusion-induced neuronal injury and inflammation in rat spinal cord and mouse microglial cells through NF-kB signaling pathway suppression [40]. BRD4 is a member of the bromodomain and extra-terminal family of proteins, which play critical roles in the regulation of gene expression through their ability to bind acetylated lysine residues on histone proteins and transcription factors. Microglial BRD4 level is increased after SCI and BRD4 suppression may attenuate M1 polarization and pro-inflammatory mediators production in microglia, through NF-kB signaling pathway suppression, enhancing functional recovery after SCI [41]. TRIM52 is a member of the tripartite motif family of proteins, which are involved in various cellular processes, including innate immunity, antiviral defense, and protein degradation. TRIM52 has been observed to activate the NF-kB signaling pathway by promoting IkBα ubiquitination, thereby regulating LPS-induced microglial cell activation and the inflammatory response, after SCI [42]. CARD6 is a member of the CARD family of proteins, which play important roles in regulating cell death and inflammation. CARD6 knockout mice exhibited a stronger inflammatory response after SCI through the NF-kB-induced expression of TNF-α, IL-1B, and IL-6 in microglia [43].

Ubiquitin-specific proteases (USPs) are a family of enzymes involved in the regulation of protein stability and turnover within cells. They belong to the larger class of deubiquitinating enzymes (DUBs), which play a critical role in the ubiquitin-proteasome system, a major pathway for protein degradation in eukaryotic cells. Animal studies have shown that USP4 expression in microglia cells decreases after SCI, leading to microglial activation and subsequent neuronal inflammation through NF-kB by attenuating the deubiquitination of TRAF6 [44]. A study by Ge et al. found that USP13 was significantly enriched in mouse microglia. USP13 connects to, deubiquitinates, and stabilizes IkBα, thus regulating microglia/macrophage polarization during SCI [45]. In animals with SCI, DUSP19-mediated SCI-induced apoptosis and inflammation via NF-kB signaling [46].

Oxidative stress plays a pivotal role in SCI. Excessive ROS production by activated microglia contributes to the amplification of neuroinflammation in the injured spinal cord. ROS can induce oxidative damage to lipids, proteins, and DNA, exacerbating tissue injury and promoting the release of additional pro-inflammatory mediators. This creates a feed-forward loop that sustains microglia activation and perpetuates neuroinflammation after SCI. Advanced oxidation protein products are a class of oxidized proteins that are formed through reactions between proteins and ROS, particularly HOCl and chloramines. They are considered biomarkers of oxidative stress and inflammation and have been implicated in various pathological conditions. Pretreated microglial cells with advanced oxidation protein products activated NADPH oxidase, triggering excessive ROS generation which induced p38 MAPK and JNK phosphorylation, subsequently triggering nuclear translocation of NF-kB p65 to express pro-inflammatory cytokines [47]. Kaempferol is a flavonoid compound found in various plants, including tea, broccoli, grapes, and Ginkgo biloba exhibiting strong antioxidant activity by scavenging ROS and reducing oxidative damage to cells and tissues. Liu et al. showed that kaempferol suppressed the LPS-induced microglial activation. In microglia cells, the administration of kaempferol inhibits ROS generation by suppressing NADPH oxidase 4 and then reducing the expression of pro-inflammatory mediators by downregulating the p38 MAPK/JNK/NF-kB signaling pathway [48]. Tacrolimus is an immunosuppressive drug commonly used in transplantation medicine to prevent organ rejection. In vitro studies have observed that the administration of tacrolimus in hypoxia-treated primary spinal cord microglia reduced microglial activation through the suppression of TNF-a, IL-1b, and IL-6 expression and NF-kB activation [49].

Several natural compounds suppress the NF-kB signaling pathway in microglia after SCI, inhibiting neuroinflammation. Curcumin, a natural polyphenolic compound, significantly downregulates the expression levels of the NF-kB upstream regulators IkB and IkB kinase (IKK), mitigating the microglia-mediated inflammatory response after SCI [32]. It is plausible that curcumin-activated olfactory ensheathing cells may exert additional anti-inflammatory effects in the injured spinal cord environment. A recent study by Jiang et al. concluded that curcumin-activated olfactory ensheathing cells could alleviate inflammation after SCI by shifting microglial polarization from M1 to M2, mediated by the APOE/TREM2/NF-kB pathway [50]. Parthenolide is a natural compound found in Tanacetum parthenium and has been investigated for its potential therapeutic effects in SCI. Its main mechanism of action involves the inhibition of the NF-kB signaling pathway and induction of HDAC1 degradation. Gaojian et al. found that parthenolide switches M1 microglia to the M2 polarization state during SCI by downregulating the NF-kB signal pathway independently of STAT1 acetylation [31]. Dl-3-n-butylphthalide is a compound derived from celery seeds and has been investigated for its potential therapeutic effects in various neurological conditions, including SCI. In an animal study by He et al, Dl-3-n-butylphthalide was found to reduce the activation of murine microglial cells, suppress the release of inflammatory cytokines, and inhibit microglial TLR4/NF-kB expression following SCI [51]. Rosmarinic acid is a natural polyphenolic compound found in various herbs, including rosemary, basil, and lemon balm, and is known for its antioxidant, anti-inflammatory, and neuroprotective effects. It was observed to mitigate LPS-induced cytotoxicity and increase apoptosis and inflammatory injury in murine microglial cells by inhibiting the TLR4/NF-kB pathway [52]. Naringin is a flavonoid glycoside found in citrus fruits such as grapefruits and oranges. It may promote functional recovery by suppressing the inflammatory response in SCI through the PPAR-γ/NF-kB signaling pathway activity [53]. Loganin is a natural iridoid glycoside compound found in several medicinal plants. It has been found to reduce neuroinflammatory responses and enhance motor recovery after SCI by inhibiting the NF-kB/NLRP3 signaling pathway by targeting the p65 protein [54]. Gramine is an alkaloid compound found in various plants, including barley grass, oats, and bamboo grass. It has been found to suppress microglia activation and enhance motor functional recovery after SCI through the NF-kB pathway [55].

Recently, iron accumulation in the brain has been associated with increased expression of inflammatory mediators and the activation of microglia. SCI may induce intracranial iron overload, activating microglia via NF-kB signaling. Activated microglia can phagocytose iron and produce inflammatory cytokines, such as TNF-α, IL-1B, and IL-6, contributing to neuroinflammation and secondary tissue damage after SCI, leading to central pain. Treatment with an iron-chelating agent can relieve central pain resulting from SCI [56].

Immunological Effects of NF-kB Through Microglia After SCI

Moreover, NF-kB activation in microglia influences the recruitment and activation of other immune cells, such as peripheral macrophages and lymphocytes, to the site of injury. This immune response can have both beneficial and detrimental effects, as it contributes to tissue repair but can also exacerbate inflammation and tissue damage. NF-kB is an important mediator of cell adhesion molecule gene expression (such as ICAM-1 and VCAM-1), regulating the interaction among microglia and other immune cells after SCI [27].

Neurotoxic Effects of NF-kB Through Microglia After SCI

NF-kB activation can also promote the expression of anti-apoptotic genes and growth factors in microglia, promoting cell survival and proliferation. This aspect of NF-kB signaling may contribute to tissue repair and regeneration following SCI. NF-kB signaling in microglia also plays a role in modulating neuronal damage following SCI. Excessive or dysregulated NF-kB activation in microglia can contribute to neurotoxicity and neuronal death through the production of cytotoxic molecules such as nitric oxide and ROS. Autophagy plays a protective role in cervical SCI by enhancing microglia polarization toward M2 through the NF-kB pathway [57].

Several natural compounds suppress the NF-kB signaling pathway in microglia after SCI, mitigating neuronal apoptosis, facilitating the M2 microglial phenotype, and alleviating tissue damage after SCI. Nesfatin-1 is a neuropeptide derived from nucleobindin 2. Nesfatin-1 has been shown to have neuroprotective effects in different neurological conditions, including ischemic stroke, traumatic brain injury, and neurodegenerative diseases. It can promote neuronal survival, reduce apoptosis, and enhance neurogenesis and synaptic plasticity. In SCI rats, nesfatin-1 has been found to exert neuroprotective actions by promoting the activation of M2 microglia through the inhibition of the TLR4/NF-kB signaling pathway [58]. The animal study by Liang et al. concluded that the TLR4-mediated activation of the NF-kB pathway may contribute to M1 microglia-induced exacerbation of neuronal apoptosis after SCI [29]. Grape seed proanthocyanidins are a class of polyphenolic compounds found in grape seeds and are known for their antioxidant, anti-inflammatory, and neuroprotective properties. Liu et al. found that grape seed proanthocyanidins regulated microglial polarisation and prevented apoptosis in M1-polarized microglial cells through the TLR4-mediated NF-kB signaling pathway [59]. Sodium houttuyfonate is a compound derived from the roots of Houttuynia cordata, a traditional Chinese medicinal herb. In vitro, sodium houttuyfonate reduced TLR4/NF-kB expression in cultured primary microglia and decreased M1 microglial polarization and cell apoptosis in an LPS-pretreated microglia and neuron coculture system [60]. Forsythoside B is a bioactive compound derived from Forsythia suspensa, a traditional Chinese medicinal herb. By blocking NF-kB activation, forsythoside B may suppress microglial activation and effectively attenuate neuro-inflammation and secondary neuronal apoptosis after SCI through Nrf2 activation [61]. Rehmannioside A is a bioactive compound derived from Rehmannia glutinosa, a traditional Chinese medicinal herb. Daily intraperitoneal injections of rehmannioside A into SCI rats significantly enhanced M2 microglial polarization, mitigated neuronal apoptosis, and increased motor function recovery, through suppression of NF-kB signal pathway [62]. Naringin may promote functional recovery by regulating microglial polarization through the PPAR-γ/NF-kB signaling pathway activity [53]. Finally, nitidine is a natural alkaloid compound, with anti-inflammatory, antioxidant, and neuroprotective properties, found in various plant species. It was found to restrict reactive microgliosis and enhance CNS repair after SCI, significantly increasing neuronal survival and decreasing neural tissue damage. Nitidine was shown to prevent cultured microglia from LPS-induced reactive activation by regulation of NF-kB signaling pathway [63].

Macrophage extracellular traps are a recently discovered phenomenon in the context of inflammation and immune responses. Zhang et al. found that macrophages infiltrated in the SCI area could induce macrophage/microglia to differentiate into M1-like cells by releasing macrophage extracellular traps, which may be achieved partly through the LL37/P2X37/NF-kB signal pathway. The restriction of macrophage extracellular traps could effectively suppress M1 polarization and enhance function recovery [64]. FPR2 is a G protein-coupled receptor primarily expressed in immune cells, including microglia. Zhang et al. showed that the activation of FPR2 repressed M1 microglial polarization by suppressing the NF-kB signaling pathways to alleviate tissue damage and locomotor decline after SCI [65].

Role of Stem Cells

Human umbilical cord mesenchymal stem cells + ultrashort wave therapy could attenuate the inflammatory microenvironment in microglia through the NUR77/NF-kB signaling pathway [66].

Role of microRNAs

MicroRNAs (miRNAs) play a crucial role in regulating gene expression post-transcriptionally and have emerged as key regulators of various cellular processes, including microglia activation after SCI. MicroRNAs can both promote and inhibit inflammatory actions among microglial cells. miR-23b mitigates the ROS-induced injury of microglial cells via the TAB3/NF-kB signaling pathway in SCI rats [67]. Triptolide is a bioactive compound derived from the traditional Chinese medicinal herb Tripterygium wilfordii. Huang et al. suggested that triptolide suppressed microglia activation following SCI through the miR-96/IKKB/NF-kB pathway [68]. MiR-100 alleviates microglial inflammation and neuronal apoptosis and improves motor function following SCI by blocking the TLR4/NF-kB pathway [69]. Jiang et al. suggested that PI3K/AKT/NF-kB signaling pathways may be involved in the modulation of microglia by exosomal miR-124-3p in SCI rats [70]. miR-130b-5p attenuates an activated microglia-induced neuron injury by targeting TLR4. Ginsenoside Rb1 has been found to suppress TLR4/NF-kB activation and inhibit proinflammatory cytokine secretion by increasing miR-130b-5p in activated microglia [71]. Catalpol is a bioactive compound found in the root of Rehmannia glutinosa, a traditional Chinese medicinal herb, known for anti-inflammatory, antioxidant, and neuroprotective effects. Catalpol may protect the spinal cord from SCI by suppressing apoptosis, oxidative stress, and inflammatory response in microglia via the miR-142/HMGB1/TLR4/NF-kB pathway [72]. MALAT1, a long non-coding RNA, contributes to the inflammatory response of microglia after SCI through the regulation of a miR-199b/IKKB/NF-kB signaling pathway [73]. Downregulation of miR-199b deteriorates the acute SCI through the IKKB/NF-kB signaling pathway activating microglial cells [74]. Liu et al. found that hypoxic exosomal miR-216a-5p may influence microglial M1/M2 polarization through the TLR4/NF-kB/PI3K/AKT signaling cascades [75]. Finally, the overexpression of MEG3, a long non-coding RNA, restrained the M1 polarization of microglia through the HuR/A20/NF-kB axis and promoted motor function recovery and neuroinflammation relief in SCI mice [76].

Circular RNAs (circRNAs) are a class of non-coding RNA molecules that form covalently closed loops and have been implicated in various biological processes, including gene regulation. Circ-Ncam2, specifically, is a circular RNA derived from the Ncam2 gene. It has been reported to function as a microRNA sponge, sequestering microRNAs and thereby modulating the expression of their target genes. A recent study by Guo et al. observed that circ-Ncam2 participates in LPS-induced microglia activation and neuronal apoptosis via the TLR4/NF-kB pathway, acting as a miR-544-3p sponge [77].

## Conclusions

The microglial response to SCI is dynamic and multifaceted, involving a complex interplay of pro-inflammatory and anti-inflammatory processes that influence the outcome of the injury. NF-kB is a critical transcription factor involved in diverse physiological and pathological processes, making it an important target for therapeutic intervention in various diseases characterized by inflammation and immune dysregulation. The role of NF-kB in the response of microglia to SCI is complex and context-dependent. While NF-kB activation is involved in initiating and propagating the inflammatory response following SCI, it also plays a role in tissue repair and regeneration. NF-kB signaling exerts profound effects on the microglial response following SCI, influencing the inflammatory milieu, tissue damage, and potential for repair and recovery. Deactivation of the NF-kB signaling pathway suppresses the production of proinflammatory mediators in microglia after SCI. NF-kB suppression has neuroprotective effects, as it mitigates neuronal apoptosis and facilitates the M2 microglial phenotype, alleviating tissue damage after SCI. Moreover, several microRNAs play a crucial role in regulating gene expression post-transcriptionally and have emerged as key regulators in microglia activation after SCI. Targeting NF-kB-mediated pathways in microglia may offer therapeutic avenues for attenuating neuroinflammation and promoting neuroprotection after SCI.
